# A Pilot Randomized Controlled Study of Dexlansoprazole MR-Based Triple Therapy for *Helicobacter Pylori* Infection

**DOI:** 10.1097/MD.0000000000002698

**Published:** 2016-03-18

**Authors:** Deng-Chyang Wu, Chao-Hung Kuo, Feng-Woei Tsay, Wen-Hung Hsu, Angela Chen, Ping-I Hsu

**Affiliations:** From the Division of Gastroenterology (D-CW, W-HH), Department of Internal Medicine, Kaohsiung Medical University Hospital; Division of Internal Medicine (D-CW), Kaohsiung Municipal Ta-Tung Hospital; Department of Internal Medicine and Cancer Center (D-CW), Kaohsiung Medical University Hospital; Cancer for Stem Cell Research (D-CW), Kaohsiung Medical University; Division of Gastroenterology (F-WT, P-IH), Kaohsiung Veterans General Hospital and National Yang-Ming University; and Institute of Biomedical Sciences (AC), National Sun Yat-Sen University, Kaohsiung, Taiwan.

## Abstract

Dexlansoprazole MR is the R-enantiomer of lansoprazole that is delivered by a dual delayed release formulation. It is effective for symptom control of patients with gastroesophageal reflux disease. However, its efficacy in the treatment of *Helicobacter pylori* infection remains unclear. This pilot, randomized, controlled, head-to-head study was conducted to investigate whether the efficacy of single-dose dexlansoprazole MR-based triple therapy was noninferior to double-dose rabeprazole-based triple therapy in the treatment of *H pylori* infection.

Consecutive *H pylori*-infected subjects were randomly allocated to either 7-day dexlansoprazole MR-based standard triple therapy (dexlansoprazole MR 60 mg once daily, clarithromycin 500 mg twice daily, and amoxicillin 1 g twice daily) or rabeprazole-based triple therapy (rabeprazole 20 mg twice daily, clarithromycin 500 mg twice daily, and amoxicillin 1 g twice daily). *H pylori* status was assessed 6 weeks after the end of treatment.

A total of 177 *H pylori*-infected patients were randomized to receive dexlansoprazole MR-based (n = 90) or rabeprazole-based (n = 87) triple therapy. Intention-to-treat analysis demonstrated no differences between eradication rates of the 2 study groups (83.3% vs 81.6%; *P* = 0.736). Per-protocol analysis yielded comparable results (85.1% vs 81.2%; *P* = 0.497). Both groups exhibited similar frequencies of adverse events (7.8% vs 4.6%; *P* = 0.536) and drug compliance (98.9% vs 97.7%; *P* = 0.496). Multivariate analysis disclosed that the presence of clarithromycin resistance was the only independent factors predictive of treatment failure with an odds ratio of 6.8 (95% confidence interval: 1.2–37.6).

This work demonstrates that single-dose dexlansoprazole MR-based triple therapy yields a similar eradication rate as double-dose rabeprazole-based therapy. Since the pharmaceutical cost of the single-dose dexlansoprazole MR regime is lower than that of the double-dose rabeprazole regimen, dexlansoprazole-based therapy can reasonably be recommended in the first-line treatment of *H pylori* infection.

## INTRODUCTION

*Helicobacter pylori* (*H pylori*) infects more than 50% of the adult population globally. The bacterium induces chronic inflammation of gastric mucosa and leads to various gastroduodenal diseases, such as peptic ulcer, gastric adenocarcinoma, and mucosa-associated tissue lymphoma.^1,2^ Currently, *H pylori* eradication has become the standard treatment to cure peptic ulcer disease.^3,4^ This therapy is also advocated in the treatment of *H pylori*-related mucosa associated lymphoid tissue lymphoma.^5^ In regions with high incidence of gastric cancer, *H pylori* eradication is recommended for the prevention of cancer development.^6,7^

Proton pump inhibitor (PPI) is one of the key medicines in anti-*H pylori* regimens. It possesses anti-*H pylori* activity,^8^ and also increases bioavailability and activity of some antibiotics by reducing gastric acid secretion.^9^ Dexlansoprazole, an R-enantiomer of lansoprazole, is a PPI with 3 to 5 times greater maximum concentration (*C*_max_), area under the plasma concentration–time curve (AUC), and a longer elimination half-life than S-lansoprazole.^10^ Dexlansoprazole modified release (MR) is a novel PPI with a dual delayed release formulation providing 2 separate releases of medication to extend the duration of effective plasma drug concentration.^11^ The dual delayed release PPI possesses 2 types of enteric-coated granules with different pH-dependent dissolution characteristics to release an initial drug in the proximal small intestine, at a pH of approximately 5.5, followed several hours later by another drug release at distal small intestine, at a pH of ≥6.0.^12^ It is effective in improving the healing of erosive esophagitis and in the treatment of symptomatic gastroesophageal reflux disease.^13–15^ However, its efficacy in the treatment of *H pylori* infection remains unclear.

Our previous study demonstrated that esomeprazole-based triple therapy achieved a higher eradication rate than pantoprazole-based regimen.^16^ The difference in eradiation efficacies between the 2 study groups is most likely due to the more powerful acid inhibition effect of esomeprazole compared with pantoprazole.^17^ A recent cross over study documented that esomeprazole at standard dose of 40 mg once daily provides more effective control of gastric acid than standard doses of pantoprazole, lansoprazole, and rabeprazole.^17^ A comparison study of dexlansoprazole 60 mg with esomeprazole 40 mg showed that dexlansoprazole MR 60 mg achieved a greater acid control than esomeprazole 40 mg (the mean percentage of time with pH >4 between 0 to 24 hours post-dose: 58% and 48%, respectively).^18^ Since single-dose esomeprazole (40 mg daily)-based triple therapy has been shown to achieve a similar eradication rate as double-dose esomeprazole (40 mg b.d.)-based therapy,^19^ dexlansoprazole MR is potentially a promising PPI which can be used in *H pylori* eradication.

Currently, the efficacy of dexlansoprazole MR-based standard triple therapy is still lacking. We therefore conducted this pilot study to assess the eradication rate of dexlansoprazole MR-based triple therapy for *H pylori* infection, and to investigate whether the efficacy of single-dose dexlansoprazole MR-based triple therapy is noninferior to double-dose rabeprazole-based triple therapy in the treatment of *H pylori* infection.

## METHODS

### Patients

This study was a prospective, noninferiority, randomized, controlled trial. Consecutive adult patients with endoscopically proven *H pylori*-related peptic ulcer diseases or gastritis were recruited for the study. The diagnosis of *H pylori* was based on at least 2 positive results of histology, rapid urease test, and culture.^20^ Criteria for exclusion criteria were as follows: age younger than 20 years; previous *H pylori*-eradication therapy, ingestion of antibiotics or bismuth within the prior 4 weeks, presence of severe comorbidities, allergy to any of the medications used in the trial, and pregnant woman. The study protocol was approved by the Ethics Committee of the Kaohsiung Medical University Hospital (IRB number: KMUH-IRB-E (I)-20150107). It was registered as a standard randomized Clinical Trial (ClinicalTrials.gov.identifier: NCT02541786).

### Randomization and Treatment

We randomly allocated patients at a 1:1 ratio to receive either a DCA (dexlansoprazole MR [Dexilant delayed release; Takeda, Osaka, Japan] 60 mg once daily, clarithromycin 500 mg twice daily, and amoxicillin 1 g twice daily) or RCA (rabeprazole [Pariet; Eisai, Misato, Japan] 20 mg twice daily, clarithromycin 500 mg twice daily, and amoxicillin 1 g twice daily) therapy according to a computer-generated number sequence. All medicines were taken 1 hour before breakfast and dinner. The random number sequence was generated by an independent study assistant. The treatment allocation was concealed in an opaque envelope until anti-*H pylori* therapy was assigned. After informed consents were obtained from the participants, a study nurse assigned anti-*H pylori* therapies according to the treatment allocations in the envelopes.

### Study Design

All recruited patients were requested to complete a questionnaire that contained questions regarding demographic data and history of smoking, alcohol drinking, nonsteroidal anti-inflammatory drug use, and underlying diseases.

The patients were informed of the common adverse events of anti-*H pylori* therapy and were requested to record the side effects during treatment. The severity of adverse events was recorded according to a 4-point scale (none; mild; moderate; severe) system as previous description.^21^ Drug compliance was determined via pill counts. Good compliance was defined as participants taking at least 80% of eradication drugs, and poor compliance was defined as participants taking less than 80% of drugs.^22^

Because a gastric cancer presenting with an ulcerative lesion might be missed by initial biopsy at endoscopy on enrollment, a follow-up endoscopy with histological examination, urease test, and culture was performed for the patients with gastric ulcers to assess the eradication outcome and the healing status of ulcers 6 weeks following anti-*H pylori* therapy. Patients with gastritis or duodenal ulcer underwent a urea breath test to assess final *H pylori* status. The urea breath test was conducted by a staff who was blind to the eradication arm. The cutoff value of urea breath test was set at 4.8% of δ^13^ CO_2_.^23^ Cure of *H pylori* infection was defined as negative results of all histology, urease test and bacterial culture, or a negative result of urea breath test.

An antral gastric biopsy specimen was obtained for *H pylori* culture, using previously described methods.^15^*H pylori* culture was performed by rubbing the specimens on the surface of a Campy-BAP agar plate (Brucella agar + IsoVitalex + 10% whole sheep blood). Then, they were incubated at 37°C with microaerobic condition for 4 to 5 days. The results of culture were regarded as positive if at least 1 colony of gram-negative bacilli with positive oxidase, catalase, and urease tests was found. The resistance to antibiotics was assessed by E-test (AB Biodisk, Solna, Sweden), and antibiotic resistances for clarithromycin, amoxicillin, and metronidazole were considered positive if the minimum inhibitory concentration values were >1, >0.5, and >8 μg/mL, respectively.^24^

### Statistical Analysis

The primary endpoint of the study was eradication rate of *H pylori*. It was evaluated by intention-to-treat (ITT) and per-protocol (PP) analyses. ITT analysis included all participants enrolled in the study regardless of drug compliance. Participants without follow-up tests for final *H pylori* status were assumed to have been treated unsuccessfully. PP analysis only included patients with good drug compliance who received follow-up examinations for eradication outcomes. The second outcomes were the frequency of adverse events and compliance to medications. Differences in baseline characteristics, eradication rates, and adverse events between groups were determined by *χ*^2^ test or Fisher exact test, as appropriate. The Student *t* test was used for the comparison of continuous data. SPSS (version 12.0 for Microsoft Windows) were used for all statistical analyses. A *P* value of <0.05 was regarded as significant difference.

According to our previous study, the eradication rate of standard triple therapy by conventional PPI is 82%.^16^ If there is a true difference in favor of the DCA treatment of 8%, at least 176 patients are required to be 90% sure that the upper limit of a 1-sided 95% confidence interval will exclude a difference in favor of the control group of more than 8%, assuming 10% loss to follow-up.

Thirteen clinical and bacterial parameters including age, sex, smoking habit, alcohol consumption (<80 or ≥80 g/day), drug compliance, and antibiotic resistance were examined by univariate analysis to search the factors related to eradication rate. A stepwise logistic regression method was then applied to search the independent factors influencing eradication outcome.

## RESULTS

### Characteristics of the Study Groups

**FIGURE 1:**
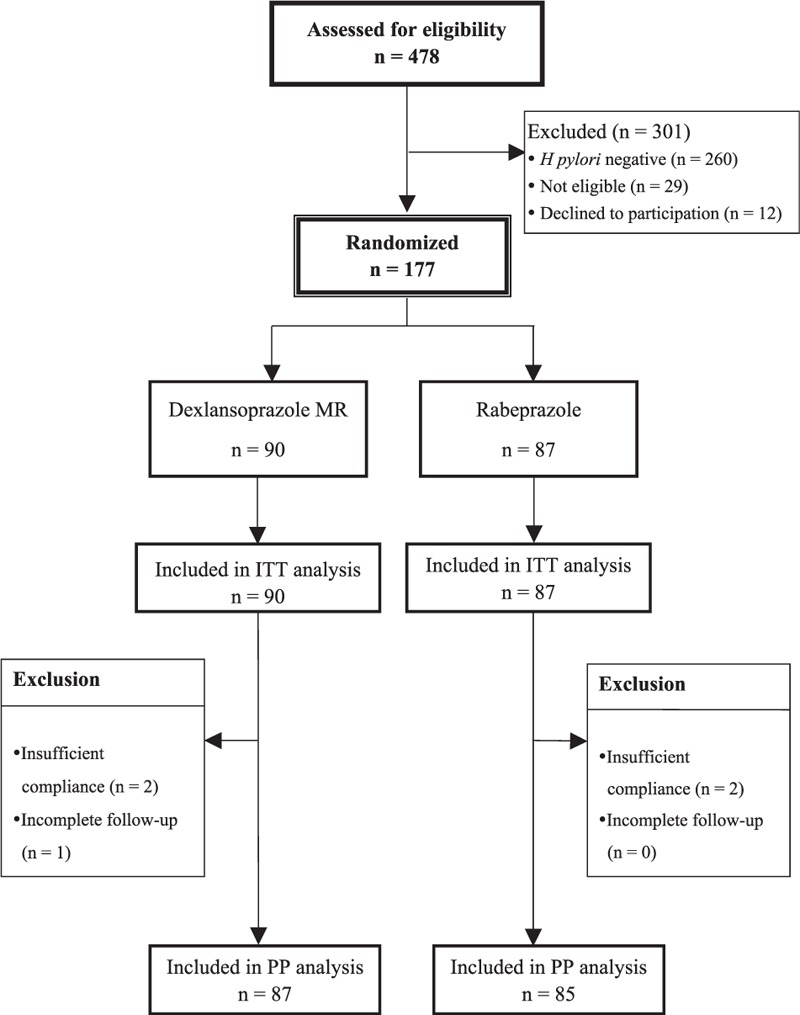
Patient disposition.

A total of 177 *H pylori*-infected participants were recruited for the study and randomly allocated to DCA (n = 90) or RCA (n = 87) therapy. The baseline demographic data and clinical parameters of the 2 treatment groups are listed in Table 1. There were no differences in all factors between groups. The flow of patients through the study is shown in Figure 1. In the recruited patients, 4 (DCA group: 2 patients; RCA group: 2 patients) with poor compliance and 1 (DCA group: 1 patient; RCA group: 0 patient) with incomplete follow-up were excluded from PP analysis.

**TABLE 1:**
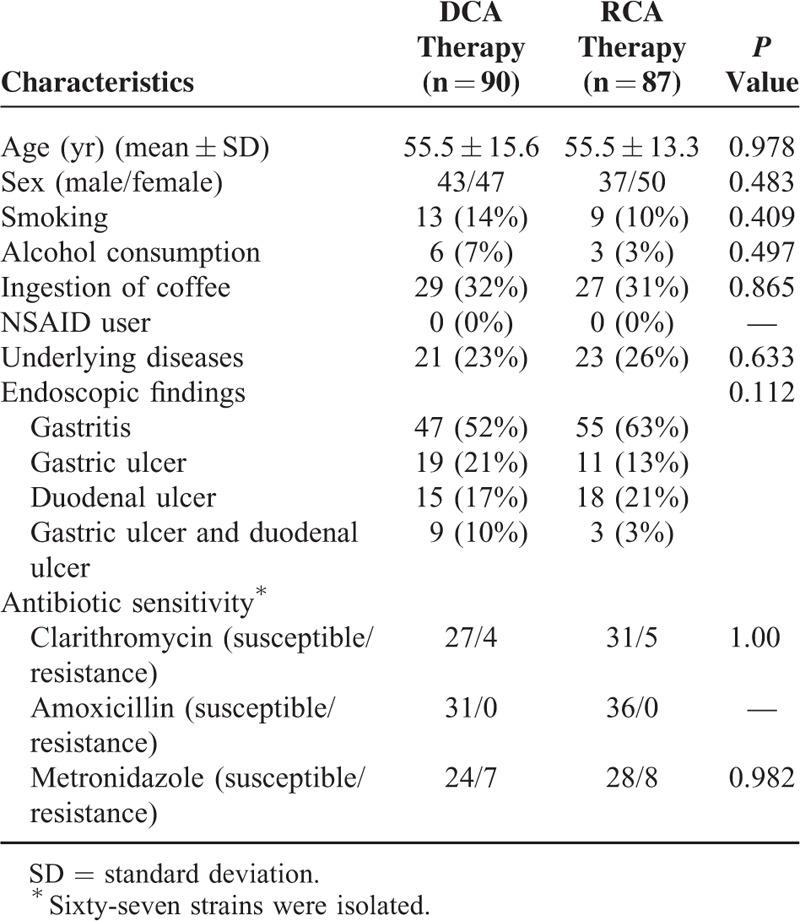
Demographic Data and Antibiotic Resistance of DCA and RCA Therapies

### Eradication of *H Pylori*

Table 2 shows the eradication rates of DCA and RCA groups. The eradication rate of DCA group was similar to that of RCA group by ITT analysis (83.3% vs 81.6%, *P* = 0.736). Additionally, PP analysis also demonstrated that the DCA and RCA groups had comparable eradication rates (85.1% vs 81.2%, *P* = 0.497).

**TABLE 2:**
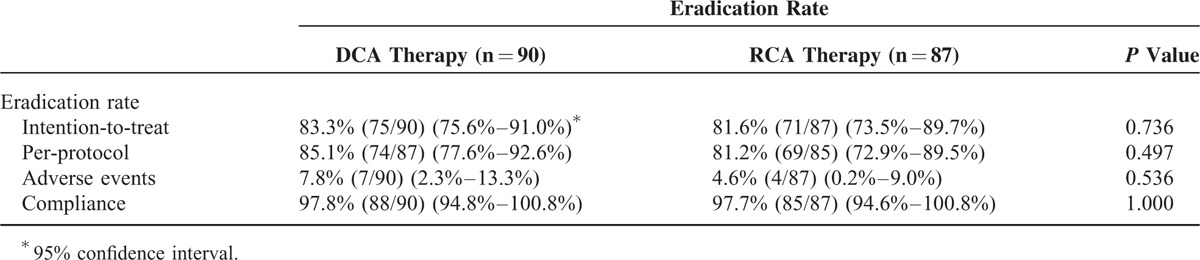
Major Outcomes of DCA and RCA Therapies

### Adverse Events and Compliances

Overall, 7.8% of the patients in the DCA group and 4.6% of those in the rabeprazole group suffered from at least 1 adverse event (*P* **=** 0.536). Table 3 lists the profiles of adverse events during eradication treatment. The 2 therapeutic groups shared similar adverse events during eradication therapy (Table 3). In the DCA group, 1 patient discontinued medicines owing to the development of skin rash. A patient in the rabeprazole group stopped treatment due to dizziness.

**TABLE 3:**
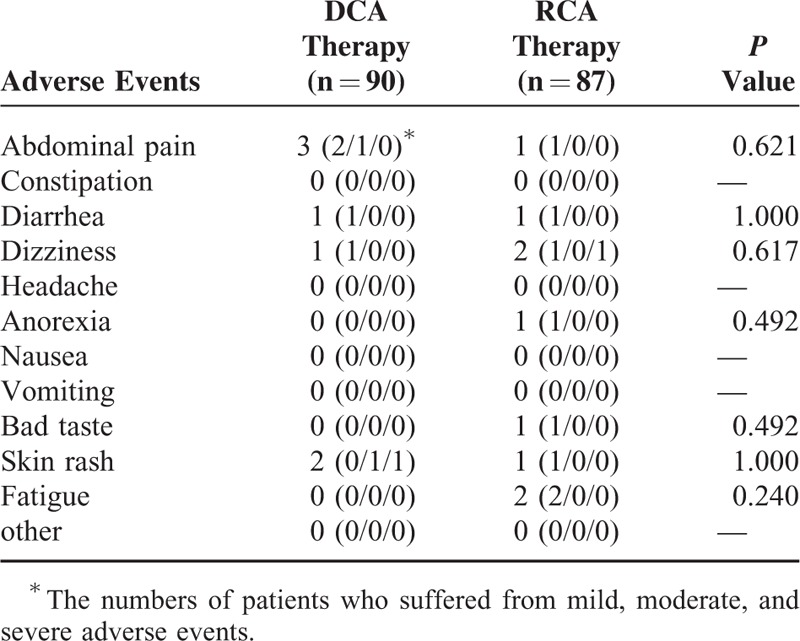
Adverse Events During DCA and RCA Therapies

The 2 treatment arms showed comparable compliance rates (97.8% vs 97.7%, *P* = 1.000). Two patients in the DCA group and another 2 in the RCA group had poor drug compliance.

### Factors Influencing Efficacy of Anti-*H Pylori* Therapy

Univariate analysis showed that clarithromycin was a factor related to the eradication outcome (*P* = 0.046; Table 4). The other factors including smoking, alcohol consumption, type of PPI, and drug compliance did not influence eradication rate. Multivariate analysis confirmed that clarithromycin resistance was an independent factor determining eradication outcome of standard triple therapy (odds ratio: 6.75; 95% confidence interval [CI], 1.21–37.64; *P* = 0.029; Table 5).

**TABLE 4:**
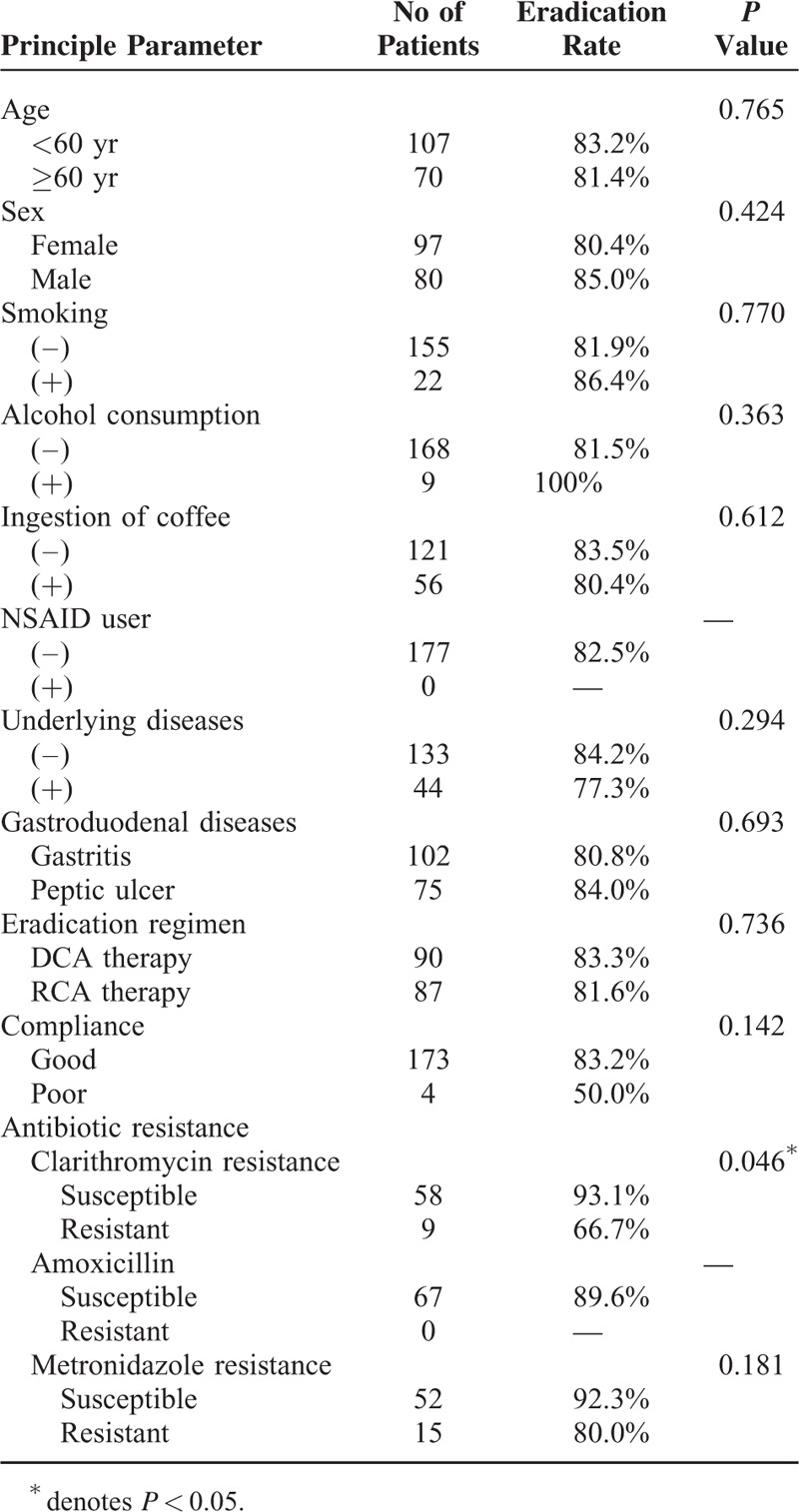
Univariate Analysis of the Clinical Factors Influencing the Efficacy of DCA and RCA Therapies

**TABLE 5:**
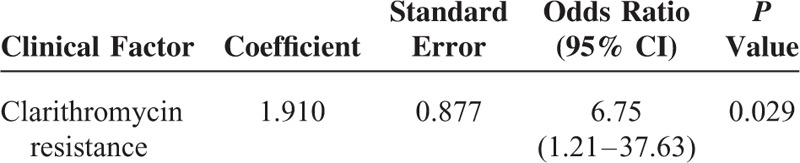
Multivariate Analysis for Independent Factors Related to Eradication Failure of Standard Triple Therapy

## DISCUSSION

In the current study, we conducted the first, head-to-head, randomized, controlled trial to investigate whether the efficacy of single-dose dexlansoprazole MR-based triple therapy was noninferior to double-dose rabeprazole-based triple therapy in the treatment of *H pylori* infection. Both ITT and PP analyses demonstrated that the eradication rate of dexlansoprazole MR-based triple therapy was similar to that of rabeprazole-based triple therapy (83.3% vs 81.6% and 85.1% vs 81.2%, respectively). Additionally, both therapies had similar frequencies of adverse events and drug compliance. The data clearly indicate that single-dose dexlansoprazole MR is noninferior to double-dose rabeprazole-base triple therapy in the treatment of *H pylori* infection.

Currently, lansoprazole 30 mg twice daily is widely used in clinical practice. In this study, single-dose of dexlansoprazole MR 60 mg daily was applied for *H pylori* eradication. In terms of cost-effective view, the pharmaceutical costs of 7-day dexlansoprazole MR- and rabeprazole-based triple therapies in Taiwan were $19.4 and $20.4, respectively. The former was cheaper than the latter. Dexlansoprazole MR-based therapies can therefore be recommended in the first-line treatment of *H pylori* infection for Taiwanese and probably most people in the world.

In the current study, the frequencies of adverse events in the dexlansoprazole-MR and rabeprazole group were 7.8% and 4.6%, respectively. The 2 therapeutic groups had comparable frequency of adverse events. Additionally, they shared similar drug compliance (97.8% vs 97.7%). In the dexlansoprazole MR group, 1 patient discontinued eradication therapy due to skin rash. On the other hand, a patient in the rabeprazole group stopped anti-*H pylori* therapy owing to severe dizziness.

The main reasons for eradication failure for *H pylori* infection include antibiotic resistance, poor compliance, *CYP2C19* genotypes, and smoking.^25–27^ In the current study, the eradication rate in the patients with clarithromycin-resistant strains was lower than that in those with clarithromycin-susceptible strains (66.7 vs 93.1%). Multivariate analysis confirmed that the presence of clarithromycin was an independent factor predictive of treatment failure (odds ratio: 6.75). Our findings were consistent with several previous studies that demonstrated clarithromcyin as a key factor influencing eradication outcome of standard triple therapy.^28–30^ The prevalence of *H pylori* strains harboring amoxicillin resistance in Taiwan ranged from 0% to 2.3% in previous reports.^19,20,23,24,31,32^ In the current trial, the rate of resistant strains to amoxicillin 0%. Since none of the *H pylori* strains were resistant to amoxicillin, assessing the impact of amoxicillin resistance on eradication outcome of standard triple therapy was precluded. It merits further studies to investigate whether amoxicillin resistance is another independent factor predicting treatment failure of the therapy.

This study was an open-label, noninferior, randomized controlled trial. We recruited 197 patients for the study assuming 8% of eradication difference and 10% loss of follow-up. The statistic power and type 1 error of this analysis were 90% and 5%, respectively. The strengths of this study included providing the data regarding antibiotic resistance. Additionally, this study investigated the impacts of antibiotic resistances on eradication rate of standard triple therapy. However, the study has several limitations. First, the current study was conducted only in a single country. The results should therefore be confirmed in other countries with different patterns of antibiotic resistances. Second, antibiotic susceptibility data were only available in some of the patients because most of the patients were referred from other gastroenterologists and culture was not performed at initial endoscopy. Additionally, the genetic factors of hosts such as *CYP2C19* genotypes determining eradication were not examined in this study. Nonetheless, this study is the pilot study to investigate the eradication rate of dexlansoprazole MR-based triple therapy. In addition, the study confirms that dexlansoprazole MR- and conventional PPI-based triple therapies have comparable eradication rates for *H pylori* infection.

In conclusion, our study demonstrates that dexlansoprazole MR-based triple therapy can achieve a similar eradication rate as rabeprazole-based therapy. Since the cost of the single-dose dexlansoprazole MR (60 mg) regimen is lower than that of the double-dose rabeprazole (20 mg) regimen, single-dose dexlansoprazole-based triple therapy can reasonably be recommended for the first-line eradication of *H pylori*.
